# Protocol for a national, mixed-methods knowledge, attitudes and practices survey on non-communicable diseases

**DOI:** 10.1186/1471-2458-11-961

**Published:** 2011-12-30

**Authors:** Alessandro R Demaio, Otgontuya Dugee, Gombodorj Amgalan, Elena Maximenco, Adiya Munkhtaivan, Silke Graeser, Tine Kryger, Janchiv Oyunbileg, Pekka Jousilahti, Maximilian De Courten, Palam Enkhtuya

**Affiliations:** 1 Copenhagen School of Global Health, University of Copenhagen, Copenhagen, Denmark; 2 Public Health Institute, Ministry of Health, Ulaanbaatar, Mongolia; 3 EPOS Health Management, MCA-Mongolia Health Project, Ulaanbaatar, Mongolia; 4 Millennium Challenge Account, Mongolia Health Project, Ulaanbaatar, Mongolia; 5 National Institute for Health and Welfare, Helsinki, Finland

**Keywords:** Non-Communicable Diseases, KAP survey, Knowledge, Attitudes, Mongolia, Injuries

## Abstract

**Background:**

Mongolia is undergoing rapid epidemiological transition with increasing urbanisation and economic development. The lifestyle and health of Mongolians are changing as a result, shown by the 2005 and 2009 STEPS surveys (World Health Organization's STEPwise Approach to Chronic Disease Risk Factor Surveillance) that described a growing burden of Non-Communicable Diseases and injuries (NCDs).

This study aimed to assess, describe and explore the knowledge, attitudes and practices of the Mongolian adult population around NCDs in order to better understand the drivers and therefore develop more appropriate solutions to this growing disease burden. In addition, it aimed to provide data for the evaluation of current public health programs and to assist in building effective, evidence-based health policy.

**Methods/design:**

This national survey consisted of both quantitative and qualitative methods. A quantitative household-based questionnaire was conducted using a nationally representative sample of 3854 rural and urban households. Participants were selected using a multi-stage cluster sampling technique in 42 regions across Mongolia, including rural and urban sites. Permanent residents of sampled households were eligible for recruitment, if aged between 15-64 years. This quantitative arm was then complemented and triangulated with a qualitative component: twelve focus group discussions focusing on diet, exercise and alcohol consumption. Discussions took place in six sites across the country, facilitated by local, trained health workers. These six sites were chosen to reflect major Mongolian cultural and social groups.

**Discussion:**

KAP surveys are well represented in the literature, but studies that aim to explore the knowledge, attitudes and practices of a population around NCDs remain scarce. This is despite the growing number of national epidemiological surveys, such as STEPS, which aim to quantify the burden of these diseases but do not explore the level of population-based awareness, understanding, risk-perception and possible motivation for change. Therefore this paper will contribute to building a knowledge base of NCD KAP survey methodology for future use in epidemiology and research worldwide.

## Background

The global increase of Non-Communicable Diseases (NCDs) in low- and middle-income countries, represents a major challenge to health services as well as social and economic development [1]. In order to set quantifiable goals and priorities for reducing NCDs, countries must collect local data on disease and risk burden as well as the knowledge and attitudes of the population. This information is crucial in order to assist with developing targeted NCD programs and policies, and in evaluating current public health interventions [2].

Mongolia is a lower-middle income country with a GDP of approximately $3600US and a population of about 2.6 million [3]. Today, more than half the total population lives in urban settings as a result of increased internal migration following transition to a free market economy in the 1990s [4]. The exact health effects and outcomes of this transition process continue to be poorly understood, as are the levels of awareness and perceptions around the health effects of this social change.

This epidemiological and health transition involving social, economic, political, cultural and health change is happening at such a fast pace that neither the Mongolian government, nor regional governments have the capability to monitor, analyse and report this process and its health effects in a timely manner to implement evidence-based prevention [5].

**Figure 1:**
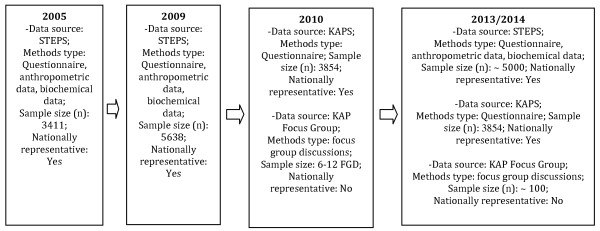
**Major Recent NCD Epidemiological Surveys in Mongolia**.

In 2005, the World Health Organization in partnership with the Mongolian National Public Health Institute conducted the first national STEPS or STEPwise Approach To Chronic Disease Risk Factor Surveillance Survey (Figure 1). With more than 3000 Mongolians sampled from across the country, this bank of biochemical and anthropometric data formed a basis for NCD epidemiology, control and prevention strategies [6]. In 2009 the STEPS survey was repeated. One concerning outcome of this research was the recognition of the rapid and unprecedented increase in NCDs which is currently occurring [2]. This group of diseases contribute a great burden of morbidity and mortality to the country where the current leading causes of death are cardiovascular disease, cancers and injuries [7]. STEPS provided important information related to practices and burden of NCDs but the surveys did not probe knowledge and attitudes towards NCDs and related risk factors. Therefore, STEPS represented one important piece of the puzzle, but not the complete public health picture.

Thus, a Knowledge, Attitudes and Practices Survey (KAPS) on NCDs complements existing STEPS data and allows public health planners and policy-makers to link and triangulate knowledge, attitudes and practices with disease and risk factor outcomes.

In 2010 the National Mongolian Knowledge, Attitudes and Practices Survey on NCDs was conducted amongst the adult Mongolian population. It aimed to identify and explore influences on NCD risk and burden. Despite KAP surveys having been used in public health for over 40 years, there continue to be very limited examples in the literature of their use in NCD epidemiology and policy. The 2010 KAP survey was a collaboration of the Mongolian National Institute of Public Health, the Copenhagen School of Global Health, EPOS Health Management and the National Institute for Health and Welfare, Finland.

The data from this survey is yet to be analysed, but is expected to serve as a valuable public health and policy tool, triangulating the major findings of the recent national STEPS surveys and acting as an evaluation tool for the Millennium Challenge Account (Mongolia) Health Project [6]. In addition, it aims to provide data to improve national NCD prevention strategies as well as existing programs aimed at early detection and screening of hypertension, diabetes, breast and cervical cancer.

### Study aim

To assess and describe the knowledge, attitudes and practices of the Mongolian adult population on Non-Communicable Diseases and injuries in order to develop evidence-based NCD prevention policies and public health interventions. In addition, to triangulate and explain the recently observed increase in NCDs in Mongolia, as was made evident by the 2005 and 2009 STEPS surveys [6].

## Methods/design

### Setting and population

This mixed-methods survey consisted of two parts.

The first, a household-based, door-to-door questionnaire was conducted in 42 regions of Mongolia, including 21 rural sites and 21 sites in the capital, Ulaanbaatar. The questionnaire was implemented across five age-stratifications, as disaggregated analysis is planned (15-24 yrs, 25-34 yrs, 35-44 yrs, 45-54 yrs and 55-64 yrs). Only permanent residents of the households sampled were eligible for recruitment, aged between 15 and 64 years.

This was complemented and triangulated by six to eight Focus Group Discussions (FGDs). The FGDs were implemented in six locations within Mongolia. These sites included:

- Urban affluent populations (national capital);

- Urban poor populations (national capital);

- Northern rural suburban;

- Northern rural remote;

- Southern rural suburban;

- Southern rural remote populations.

This qualitative arm did not aim to be nationally representative, but purposefully sampled populations with varying cultures and demography from the Russian-Mongolian border, the Chinese-Mongolian border and the urban environment of the Mongolian capital, Ulaanbaatar.

### Questionnaire and focus group discussions

The quantitative arm of this research was a field-worker-administered, household-based questionnaire undertaken on a nationally-representative sample (see Appendix 1 for full questionnaire). The questionnaire included 106 questions and took 25-40 min to complete, with some optional sections completed by only specific demographic groups (e.g. breast cancer in women). Following administrative questions were sections covering broad NCD risk factor awareness and general risk perception pertaining to:

i. Alcohol use;

ii. Tobacco use;

iii. Diet and nutrition;

iv. Physical activity and exercise;

v. Stress management;

vi. Weight and overweight;

vii. General attitudes towards other NCD risk factors.

Then focused sections investigated public knowledge and attitudes towards:

i. Cardiovascular disease (incl. stroke);

ii. Diabetes mellitus;

iii. Breast and cervical cancers;

iv. Road traffic injuries;

v. Mental health.

Finally, these thematic sections were followed by questions on the participant's demography, living conditions, level of education and affluence.

Given the sampled population was different from those sampled in the STEPS surveys, the questionnaire also included a limited number of important questions probing practices relating to chronic diseases, smoking status, alcohol consumption, nutrition and disease-detection. This will enable further in-depth analysis of the knowledge and attitudes and how these related to specific behaviours or practices.

Reflecting the paucity of NCD-related KAP surveys available, questions for this survey were adapted from a range of sources including:

- International clinical guidelines and policy documents; [8,9]

- Established clinical assessment tools; [10]

- KAP survey construction guides; [11]

- Existing KAP surveys on topics related to NCDs; [12,13]

- Existing KAP surveys on other health topics [14-17].

The second arm of this survey was 6-12 focus group discussions across 6 sites exploring the topics of diet, exercise and alcohol consumption in more detail (Table 1).

**Table 1:** Topics of Focus Group Discussions Question Guides

Topics of discussions	Directions of discussions
Nutrition	1. Knowledge of the role of fruit and vegetable consumption as a part of a healthy diet and nutrition; 2. Factors influencing fruit and vegetable consumption (social, economic, geographic, access); 3. Knowledge of negative and positive influences of fruit and vegetable consumption among the population and availability of information related to it; 4. Direct and indirect dietary sources of salt and knowledge the health impacts of salt consumption.

Physical activity	1. Understanding of what is physical activity; 2. Positive and negative health impacts of being physically active and inactive; 3. Awareness of types of physical activity.

Alcohol consumption	1. Thoughts and knowledge of appropriate alcohol consumption; 2. Factors influencing excessive alcohol consumption; 3. Knowledge of the negative impacts and health risks of alcohol consumption.

45 local trained health workers from the National Public Health Institute administered the two-part survey. These field workers were trained in questionnaire implementation and focus group discussion facilitation by the expert working group (EWG) of national and international researchers.

### Sampling methods and sample size

For the questionnaire, sample size was calculated for a nationally-representative sample and to reflect the need for disaggregated data analysis by age, gender and urbanicity. The calculation of the representative sample of the population was estimated considering a 95% confidence level. The baseline indicator was assumed at 50% and the acceptable margin of error of 6%. A complex sample design effect of 1.3 and equal representation of gender and age in each groups (5 groups in 10 years interval) was adopted, based on methodologies used for STEPS. Therefore, a sample size of 3,468 participants was determined. Based on previous survey experience an assumed non-response rate of 10% was adopted, resulting in an overall n = 3,854.

Participants for the quantitative research component were then recruited using a multi-stage cluster sampling technique across 42 locations. Sampling included four stages (Table 2):

**Table 2:** Participants for the quantitative research component were then recruited using a multi-stage cluster sampling technique across 42 locations. Sampling included four stages

Primary Sampling Unit	Based on proportional population to size (PPS) sampling, each of the 21 clusters was selected from 137 Khoroos (suburbs) in urban and 324 Soums (regions) in rural areas respectively;
Secondary Sampling Unit	For each Khoroo and Soum, 2 family centres (groups of ± 1000 households) and Baghs (sub-regions) were selected respectively using PPS;

Tertiary Sampling Unit	Based on the list of households from the selected secondary sampling units, 82-84 households were selected using simple random sampling to make a final sample;

Participants	One person aged 15-64 years in the selected household was selected at random. These were selected at random using a predefined, paper-based algorithm.

Focus group discussions of six to eight participants each, took place in six sites across the country. These sites were chosen in order to sample major known Mongolian cultures and social groups believed to likely influence the knowledge, attitudes and practices being studied. Purposive and snowball sampling were undertaken in the field to select focus group discussion participants. Trained health workers completed this sampling process in the field.

In addition to a group facilitator, each focus group had a trained scribe/observer. Discussions were also recorded and verbatim transcriptions made in Mongolian. These transcriptions were then complemented with notes, comments and clarifications from the scribe/observer.

### Research rigour and quality control

In order to ensure scientific rigour in this research, a number of processes were included in questionnaire and FGD planning and methodologies.

1. Translation and Back Translation: The questionnaire was initially developed in English by the EWG of local and international researchers. Once completed, professional Mongolian translators with experience in medicine and public health translated the questionnaire into Mongolian (single national language). Mongolian speaking members of the EWG then checked the Mongolian version of the questionnaire. It was then back translated into English and again checked by the EWG.

2. Peer Review: At four stages during the questionnaire development, a peer-review adapted online Delphi process was undertaken drawing on expertise from all international research partners.

In addition, during the final stages of development, a 15 person local expert panel from the Mongolian Ministry of Health and National Public Health Institute reviewed the questionnaire and question-guide in detail, analysing all sections question-by-question. The experts scrutinised questions pertaining to specific NCD-related health behaviours or practices. This panel consisted of clinicians, public health specialists and researchers.

3. Pretesting: a pretesting process was undertaken using a Cognitive Interviewing technique with both clinicians and lay people [18]. This process provided crucial and detailed insight into the fluency and interpretation of all questions.

4. Piloting: occurring in a controlled setting during the training of the 45 field workers, each was asked to administer the questionnaire six times to one another. Members of the EWG were on hand to take comments and record problems or observations. Mock focus group discussions were also run within the field worker training exercises. This allowed for pretesting of the FGD question guides and familiarisation of the methods.

### Planned analysis

For quantitative data, the prevalence and measures of central tendency of NCD risk factors, knowledge, attitudes and behaviours will be estimated. Population and sample weighting will be applied. Expected outcome measures (prevalence and mean variance) and differences between groups (age, gender and urban/rural groups) will be calculated with 95% confidence intervals. Sampling error, which could potentially affect the accuracy of the results of the survey, will be estimated from the standard error of variables. Multivariate regression analysis will be used to explore the relationship between knowledge, risk attitudes and disease or demographic parameters.

Consistent with a mixed-methods research approach, qualitative data will be used to triangulate quantitative data collected through the questionnaire. Analysis of qualitative data will be done using Directed Qualitative Content Analysis, conducted on the transcriptions taken from the FGD. In addition, comments, observations and concerns from the observer/scribe will be used to triangulate the transcriptions, affording greater opportunity for accuracy and interpretation. This process will be completed in Mongolian by researchers of the EWG and then translated into English.

### Ethics approval

This study was conducted according to the principles of the Helsinki declaration. The Mongolian National Ministry of Health's Medical Ethical Committee approved the study on the 06 October 2010.

**Table 3:** Survey Timeline

	Aug-Oct 10	Nov-Dec 10	Jan-Mar 11	Apr-Aug 11	Sept 11-Sept 12
Questionnaire and Question Guide Development					

Field Worker Training					

Data Collection					

Data Entering and Transcription					

Data Analysis					

Reporting					

The 2010 KAP survey, including data analysis and reporting is to be completed between August 2010 and September 2012 (Table 3).

## Discussion

This unique national survey involved a team of researchers from more than 5 countries, and the development of its methods and the questionnaire presented many challenges.

One hurdle was designing a new KAP questionnaire with few previous examples upon which to base our work. Despite more than 40 years since KAP surveys were first devised, little has been published with regards to NCDs and KAP surveys. A paucity of KAPS designed or implemented in low and middle-income countries further compounded this challenge. Our strategy for solving this was two-fold. Firstly, we widened our search scope and included current clinical guidelines, established clinical assessment tools such as the WHO AUDIT and KAP surveys in related or analogous fields - for example for HIV or TB [10]. This proved fruitful and afforded a greater basis upon which to devise our methods and questionnaire. We also utilised proven components from the STEPS methodology, including sampling frames and solutions to field-worker logistics. The second important mechanism in solving this issue was to have a multi-disciplinary working group, which included epidemiologists, policy-analysts, psychologists and medical doctors. This diversity allowed us to bring skills and experience from a range of backgrounds to more efficiently create a rigorous NCD KAPS.

Our global and multidisciplinary team of partners, working to design and implement this survey was indeed a strength but also presented challenges for communication regarding questionnaire and methodology formulation. Utilising an adapted email-based Delphi approach, information was able to be collected from all parties to a central facilitator in a timely fashion. This feedback was then summarized and disseminated in a second round to all involved. Once agreed upon, decisions could be implemented efficiently. The facilitator, or gatekeeper for information, was usually Mongolian and this ensured the process was inclusive and transparent. We found this adapted Delphi approach very useful and effective, minimising the need for international travel and catalysing the planning processes.

This survey was not only large by sample size, but was implemented on a daunting geographic scale. Mongolia is the second-largest landlocked country in the world, so ensuring a nationally representative sample presented enormous logistic challenges. Timing was also limited as winter temperatures can drop to -40°C and a series of delays resulted in a rapidly closing implementation window. This was again, where having strong local research leaders became crucial. For the data collection, we were able to enrol and train 45 health workers from the National Public Health Institute. This number allowed us to send more than 20 data collection teams to the field at once. Similarly, logistic challenges were only overcome with local knowledge and experience. In this light, the leadership and support of local partners was essential and allowed for all data to be collected nationally within just 2 weeks.

One of the strengths of our methodology and approach, reported by many of the fieldworker team-leaders, was the high level of preparedness experienced by them and their teams upon reaching the field. This not only saved time, but also ensured greater scientific rigour for our survey. Thorough pretesting and piloting before departing for the field meant fieldworkers felt familiar with the sampling and research methods, as well as the questionnaire and discussion guides. We were also able to ensure that the questionnaire, designed to be read like a script, was fluent and consistent for both the fieldworker and the interviewee. This pretesting and piloting process was completed under the supervision of members of the EWG. Importantly, this allowed the questionnaire and methods to be improved as issues arose and before implementation began.

Finally, KAP surveys are well represented in the literature but studies that aim to explore the knowledge, attitudes and practices of a population around Non-Communicable Diseases and injuries remain scarce. This is despite the growing number of national surveys, such as the WHO STEPS, being implemented worldwide which aim to quantify the burden of these diseases but do not explore population-level awareness, understanding, risk-perception or motivation for change. Therefore, in order to understand NCDs from a population perspective and build evidence-based public policy and interventions aimed at addressing NCDs, KAP surveys such as this will prove increasingly important (Additional File 1).

## Competing interests

The authors declare that they have no competing interests.

## Authors' contributions

AD and TK drafted the manuscript. AD, OD, GA, EM, SG and MdC participated in the design of the study. OD and JO obtained funding for the project. All authors read, commented, and approved the final version of the manuscript.

## Pre-publication history

The pre-publication history for this paper can be accessed here:

http://www.biomedcentral.com/1471-2458/11/961/prepub

## Supplementary Material

Additional file 1 **KAP Questionnaire (DOCX 78 kb)**. Click here for file
