# Supplementary material for: Protocol for a national, mixed-methods knowledge, attitudes and practices survey on non-communicable diseases
Source: BMC Public Health. 2011 Dec 30;11:961. doi: 10.1186/1471-2458-11-961 (PMC3280340; doi:10.1186/1471-2458-11-961)
Supplement: Additional file 1 — KAP Questionnaire (DOCX 78 kb). [file 1471-2458-11-961-S1.DOCX]

**NATIONAL KNOWLEDGE, ATTITUDES AND PRACTICES SURVEY ON NON-COMMUNICABLE DISEASES**

| **1. Survey Information (I)** | | | | |
| --- | --- | --- | --- | --- |
| Location and Date | | Response | |  |
| 1 | Cluster number | └─┴─┴─┘ | | I1 |
| 2 | Household number | └─┴─┴─┘ | | I2 |
| 3 | Interviewer number | └─┴─┴─┴─┘ | | I3 |
| 4 | Date of completion of the instrument | └─┴─┘└─┴─┘└─┴─┴─┴─┘  dd mm year | | I4 |
| 5 | Area | Urban 1  Rural 2 | | I5 |
| 6 | Name and code of aimag/ city | ............................................ | | I6 |
| 7 | Name and code of soum / district | .............................................. | | I7 |
| 8 | Name and code of bag/ khoroo | ................................................ | | I8 |
| *Answer to the questions below (9-14) at the end of the interview.*  *🡺****Now go to the next page and ask questions related household characteristics*** | | | | |
| 9 | Result of HH interview | Completed | 1 | I9 |
|  |  | Refused  Not at home  HH not found /destroyed | 2  3  4 |  |
|  |  | Other (specify)________________ 5 | | I10 |
| 10 | Total number of household members living together | **└─┴─┴─┘** | | I11 |
| 11 | Total number of children (aged 0-14) | └─┴─┘ | | I12 |
| 12 | Data entry clerk | └─┴─┘ (Signature) | | |
| 13 | Data editor | └─┴─┘ (Signature) | | |
| 14 | Supervisor | └─┴─┘ (Signature) | | |

| **2. General Knowledge and Attitudes related to Non- Communicable Diseases (GK)** | | | | |
| --- | --- | --- | --- | --- |
| **Question** | | **Response** | | **QN** |
| *I would like to ask you some questions about health, focusing on a group of diseases called non-communicable diseases or NCDs.*  *Please tell me if the following is true, false or you don’t know:* | | | | |
| 15 | A non-communicable disease is one that cannot be spread between people? | True 1  False 2  I don’t know 3 | | GK1K |
| *Non-communicable diseases are a group of diseases that include heart disease, diabetes and cancers. These are diseases that you cannot catch, but that develop from a number of factors, usually over a long time.*  *Please tell me if you strongly disagree, disagree, agree or agree strongly with the following sentences:* | | | | |
| 16 | Non-communicable diseases are less dangerous than infectious diseases. | \| Strongly disagree \| Disagree \| Agree \| Strongly agree \| \| --- \| --- \| --- \| --- \| \| 1 \| 2 \| 3 \| 4 \| | | GK2A |
| 17 | Non-communicable diseases are common amongst Mongolians. | \| Strongly disagree \| Disagree \| Agree \| Strongly agree \| \| --- \| --- \| --- \| --- \| \| 1 \| 2 \| 3 \| 4 \| | | GK3A |
| 18 | Have you ever been told you have or had any of the following:  ***(Nr. 4 and 5 to be addressed only to women)***  *If person is unsure, ask question again. If still unsure, answer “no”.* | High blood pressure 1  Diabetes 2  Heart attack or stroke 3  Breast cancer 4  Cervical cancer 5 | Yes 1 No 2  Yes 1 No 2  Yes 1 No 2  Yes 1 No 2  Yes 1 No 2 | GK4 |
| *I would now like to ask you about smoking and tobacco.* | | | | |
| 19 | Regarding tobacco smoking, please choose which one applies to you. | I have never smoked 1  I’m a Previous/Ex-smoker 2  I am a current smoker, but not every day 3  I am currently a daily smoker3 | | GK5P |

| **3. Knowledge, Attitudes and Practices on NCD Behavioural Risk Factors (RF)** | | | | | | | | |
| --- | --- | --- | --- | --- | --- | --- | --- | --- |
| **tobacco use** | | | | | |  | | *QN* |
| 20 | | Does smoking affect your own health? | | | Yes 1  No 2 (skip next 5 questions)  I don’t know 3 (skip next 5 questions) | | | *RF1K* |
| 21 | | How much do you have to smoke for it to harm your health?  (read) | | | Any smoke harms your health 1  You must smoke at least once a week 2  Only daily smoking is harmful 3  Only a packet of cigarettes or more per day is harmful 4 | | | *RF2A* |
| 22 | | Does smoking harm your lungs? | | | Yes 1  No 2 (skip next)  I don’t know 3 (skip next) | | | *RF3K* |
| 23 | | If yes: “Is it harmful or very harmful?” | | | Harmful 1  Very harmful 2 | | | *RF4A* |
| 24 | | What about your heart, does smoking harm your heart? | | | Yes 1  No 2 (skip next)  I don’t know 3 (skip next) | | | *RF5K* |
| 25 | | If yes: “Is it harmful or very harmful?” | | | Harmful 1  Very harmful 2 | | | *RF6A* |
| ***🡪🡪🡪****Now, a question about smoking near other people.* | | | | | | | | |
| 26 | | Do you think smoking around others could affect their health? | | | Yes 1  No 2  I don’t know 3 | | | *RF7K* |
| *How about smoking at home.* | | | | | | | | |
| 27 | | Do you mind if people smoke in your home?  You don’t mind, you do mind but allow it or you don’t allow it. | | | Don’t mind 1  I do mind but I allow it 2  I don’t allow it 3 | | | *RF8A* |
| *What about work?* | | | | | | | | |
| 28 | | How important is it to you to have a smoke-free workplace?  Is it not important, quite important, important or very important. | | | \| Not at all \| Moderately important \| Important \| Very  important \| \| --- \| --- \| --- \| --- \| \| 1 \| 2 \| 3 \| 4 \| | | | *RF9A* |
| 29 | | Has a health worker ever talked to you about the harms of smoking? | | | Yes 1  No 2 | | | RF10P |
| **alcohol use**  *The next few questions will be about alcohol; including beer, vodka, wine, airag and whisky* | | | | | | | | |
| 30 | | Do you agree strongly, agree, disagree or disagree strongly with this statement?  In general, when Mongolians drink alcohol, they tend to drink large amounts at once.  *(can repeat once)* | | | \| Strong agree \| Agree \| Disagree \| Strongly disagree \| \| --- \| --- \| --- \| --- \| \| 1 \| 2 \| 3 \| 4 \| | | | RF11A |
| **31** | | On which of the following occasions would Mongolians commonly drink large amounts of alcohol? | | | \| Celebrations  Customs or traditions  Drinking with friends or family  After receiving your income  There is usually no special reason \| Yes 1  Yes 1  Yes 1  Yes 1  Yes 1 \| No 2  No 2  No 2  No 2  No 2 \| \| --- \| --- \| --- \| | | | RF12P |
| *And now regarding your own drinking.* | | | | | | | | |
| **32** | Do you ever drink any alcohol? | | | Yes 1  No 2 (skip next 4 **🡪**) | | | | *RF13P* |
| **33** | | I will now give you 5 common reasons for dinking alcohol.  Please rank the following reasons starting with the most common reason you drink, to the least common? | | | To forget your problems 1 ___  To relax 2 ___  For enjoyment 3 ___  To drink with friends 4 ___  To help digestion 5 ___ | | | RF14P |
| *Next, I would like to ask you about drinking at certain times of the day.* | | | | | | | | |
| 34 | | In the past month, have you ever had an alcoholic drink between waking up in the morning and midday? | | | Yes 1  No 2 | | | *RF15P* |
| The next question is a little bit personal, but we ask everyone. Don’t feel embarrassed. | | | | | | | | |
| 35 | | Have you ever thought there is a need to reduce the amount of alcohol you drink? | | | Yes 1  No 2 | | | *RF16P* |
| The next question is about driving. | | | | | | | | |
| 36 | | Do you drive a car? | | | Yes 1  No 2 (skip next) | | | *RF17P* |
| 37 | | Have you ever driven whilst influenced by alcohol? | | | Yes 1  No 2 | | | *RF18P* |
| **🡪🡪🡪**Finally, | | | | | | | | |
| 38 | | Have you ever been advised by health workers about the harms of drinking alcohol? | | | Yes 1  No 2 | | | *RF19P* |
| **Diet**  *Changing focus, let’s talk about a healthy diet* | | | | | | | | |
| 39 | | In general, how important is it for people to eat fruit and vegetables every day?  Not important, moderately important, important or very important? | | | \| Not \| Moderately important \| Important \| Very important \| \| --- \| --- \| --- \| --- \| \| 1 \| 2 \| 3 \| 4 \| | | | *RF20A* |
| Ok, I would like to ask you about your diet | | | | | | | | |
| 40 | | How important is it to you to eat fruit everyday?  Not important, moderately important, important or very important? | | | \| Not \| Moderately important \| Important \| Very important \| \| --- \| --- \| --- \| --- \| \| 1 \| 2 \| 3 \| 4 \| | | | *RF21A* |
| *Now a question about vegetables. For this question, we are only asking about green and coloured vegetables. Not potatoes, grains or rice.*  *Green and coloured vegetables. Not potatoes, grains or rice.* | | | | | | | | |
| 41 | | How important is it to you to eat vegetables everyday?  Not important, moderately important, important or very important? | | | \| Not \| Moderately important \| Important \| Very important \| \| --- \| --- \| --- \| --- \| \| 1 \| 2 \| 3 \| 4 \| | | | *RF22A* |
| *Now, about the diet of Mongolians generally. Many Mongolians don’t eat a lot of fresh fruits and vegetables.* | | | | | | | | |
| 42 | | What do you think are the main reasons Mongolians don’t eat more fruits and vegetables?  (Open ended, m*ore than one ok, do not prompt))*  Only: “Anything else?” | | | Price 1  Availability 2  Taste 3  Lack of knowledge 4  Cultural/Dietary Customs 5  Distrust for imported foods 6  Other (specify)____________________7 | | | *RF23P* |
| *I would like to ask you about animal fat that you eat: this includes fat from meat, butter and other milk products.* | | | | | | | | |
| 43 | | In general, do you think Mongolians eat too much animal fat? | | | Yes 1  No 2 (skip next) | | | *RF24A* |
| 44 | | From the following or other, what is the main reason you eat animal fat?  (read) | | | Customs and traditions 1  Taste 2  For health 3  For energy 4  Other ________________ 5 | | | *RF25P* |
| *And now some questions on salt in your diet.* | | | | | | | | |
| 45 | | Do you ever think about how much salt you have in your diet? | | | Yes 1  No 2 | | | *RF26A* |
| **46** | | Which of the following sources contributes the largest amount of salt to the daily diet of a Mongolian? (read all) | | | The salt they add themselves to food and drinks 1 From raw foods such as milk, meat and vegetables 2 From factory-made foods such as bread, sausages and biscuits 3 | | | *RF27K* |
| 47 | | How often do you add salt when cooking or eating meals?  Never, sometimes, often or always. | | | \| Never \| Sometimes \| Often \| Always \| \| --- \| --- \| --- \| --- \| \| 1 \| 2 \| 3 \| 4 \| | | | *RF28P* |
| 48 | | When you eat meals not at your own house, do you tend to find the food others cook too salty, normal or not salty enough?  This includes restaurants, friends. | | | Too salty 1  Normal 2  Not salty enough 3 | | | *RF29A* |
| 49 | | I will now read out 5 foods and I would like you to tell me if they are high, medium or low in salt: | | | \| Food \| Low \| Medium \| High \| \| --- \| --- \| --- \| --- \| \| Rice \| 1 \| 2 \| 3 \| \| White bread \| 1 \| 2 \| 3 \| \| Ketchup \| 1 \| 2 \| 3 \| \| Potato chips \| 1 \| 2 \| 3 \| \| Pickled vegetables \| 1 \| 2 \| 3 \| \| Sausages \| 1 \| 2 \| 3 \| | | | *RF30K* |
| 50 | | Now, a question about the traditional Mongolian milk tea. When preparing milk tea, do you usually add salt? | | | Yes 1  No 2 (skip next) | | | *RF31P* |
| 51 | | Approximately how many cups of milk tea do you drink each day? | | | ______  Less than 1 each day 1 | | | *RF32P* |
| 52 | | In general, do you think Mongolians consume too much salt? | | | Yes 1  No 2 | | | *RF33A* |
| **Exercise**  *Let’s talk about exercise and health now:* | | | | | | | | |
| 53 | | In your opinion how often should a person do exercise to stay healthy?  *(List)* | | | Monthly 1  Twice a month 2  Once a week 3  1-4 times per week 4  5 or more times per week 5 | | | *RF34K* |
| *The recommended amount of exercise to stay healthy is at least for 10mins, 5 times a week. But many Mongolians don’t get this much exercise.* | | | | | | | | |
| **54** | | What are the main reasons many Mongolians don’t get this much exercise? From the following options, choose one. | | | No time 1  Too expensive 2  They don’t know how 3  They don’t want to 4  They think there is not need 5  Other (specify)__________________ 6 | | | *RF35P* |
| **Stress Management**  *Now I am going to ask you about stress* | | | | | | | | |
| **55** | | From day to day, how often do you feel stressed? | | | Always 1  Often 2  Sometimes/Infrequently 3  No, never 4 (skip next) | | | *RF36A* |
| 56 | | What is currently the main cause of stress in your life?  Choose from the following list. | | | Family 1  Relationships 2  School/university 3  Work/Lack of work 4  Money 5  Health 6  Other (specify)____________________7 | | | *RF37A* |
| 57 | | In general, please list any ways that someone could reduce their own stress?  (Open ended, no prompt) | | | Exercise 1  Talk to friends/family 2  Eat 3  Talk to health workers 4  Drink some alcohol 5  I don’t know 6  Other (specify)____________________7 | | | *RF38A* |
| **High Blood Pressure**  *OK, Now let’s talk about blood pressure* | | | | | | | | |
| 58 | | | How much do you know about “blood pressure”?  Nothing at all.  You have only heard the term before but know nothing more.  You know a little about blood pressure  You are very familiar with it. | | \| Nothing at all \| I have only heard the term before \| I know a little about it \| I am very familiar with it \| \| --- \| --- \| --- \| --- \| \| 1 \| 2 \| 3 \| 4 \| | | | *RF39K* |
| *Blood pressure is the level of pressure in your blood vessels. It is measured by a doctor or other health worker.* | | | | | | | | |
| 59 | | | Do you think it is important that Mongolians have their blood pressure checked? | | Yes 1  No 2 | | | *RF40A* |
| ***(next two to be addressed only to person older than 35 years)*** | | | | | | | | |
| 60 | | | Approximately, how regularly do you think Mongolians your age should have their blood pressure checked?  Please choose either: | | It is not needed to check regularly 1  Every 5 years 2  Every 2 years 3  Once a year 4  More often than once a year 5 | | | *RF41K* |
| *But many Mongolians do not get their blood pressure checked regularly.* | | | | | | | | |
| 61 | | | What do you think is the main reason that they do not check their blood pressure regularly?  Please choose one.  (Read) | | Don’t have time 1  Don’t know where to access service 2  Don’t think it is important 3  Don’t know how 4  Didn’t know I had to 5  Others (specify)____________________6 | | | *RF42A/P* |
| *It is ok if you do not know the answer but:* | | | | | | | | |
| **62** | | | Can eating food with a lot of salt affect blood pressure? | | Yes 1  No 2 (skip next)  I don’t know 3 (skip next) | | | *RF43K* |
| **63** | | | How would eating food with a lot of salt affect you blood pressure? Would it raise or lower your blood pressure? | | Raise it 1  Lower it 2 | | | *RF44K* |
| 64 | | | Does high blood pressure can cause health problems? | | Yes 1  No 2 (Skip next)  I don’t know 3 (skip next) | | | *RF45K* |
| 65 | | | Does high blood pressure affect the following body parts: | | \|  \| Yes \| No \| I don’t know \| \| --- \| --- \| --- \| --- \| \| The brain \| 1 \| 2 \| 3 \| \| The kidneys \| 1 \| 2 \| 3 \| \| The heart \| 1 \| 2 \| 3 \| | | | *RF46K* |
| *Finally, before we move on, I would like to ask you about ways to reduce your blood pressure.* | | | | | | | | |
| **66** | | | I will now list 4 potential treatments or activities.  Please rate each of them as not effective, effective or very effective to reduce blood pressure. | | \|  \| Not effective \| Effective \| Very effective \| \| --- \| --- \| --- \| --- \| \| Medication \| 1 \| 2 \| 3 \| \| Losing weight \| 1 \| 2 \| 3 \| \| Changing your diet \| 1 \| 2 \| 3 \| \| Exercise \| 1 \| 2 \| 3 \| | | | *RF47A* |
| **Weight**  *Now we will talk about body weight* | | | | | | | | |
| 67 | | | Have you weighed yourself in the last 6 months? | | Yes 1  No 2 | | | *RF48P* |
| 68 | | | Regarding your body weight, do you feel you are:  (read all) | | Underweight 1  Normal weight 2  Overweight 3  Very overweight 4 | | | *RF49A* |
| 69 | | | How important is having a normal body weight to you?  Not important, moderately important, important or very important. | | \| Not at all \| Moderately important \| Important \| Very  important \| \| --- \| --- \| --- \| --- \| \| 1 \| 3 \| 4 \| 5 \| | | | *RF50A* |
| **Summary of RF**  *So we have talked a lot about diet, exercise, smoking and more.* | | | | | | | | |
| 70 | | | For each of he following things, please rate how harmful you think each one is for your health?  Please rate as not harmful, moderately harmful, harmful or very harmful.  (Do not prompt, but can repeat the question/rating) | | \|  \| Not \| Moderately  harmful \| Harmful \| Very harmful \| \| --- \| --- \| --- \| --- \| --- \| \| Drinking alcohol every day \| 0 \| 1 \| 3 \| 4 \| \| Eating food with lots of salt \| 0 \| 1 \| 3 \| 4 \| \| Smoking \| 0 \| 1 \| 3 \| 4 \| \| Being overweight \| 0 \| 1 \| 3 \| 4 \| \| Eating low amounts of fruit and vegetable \| 0 \| 1 \| 3 \| 4 \| \| Being physically inactivity \| 0 \| 1 \| 3 \| 4 \| | | *RF51A* | |

| **4. Knowledge, Attitudes and Practices Related to Cardiovascular Diseases (CD)** | | | |
| --- | --- | --- | --- |
| **Question** | | **Response** | **Code** |
| *I would now like to ask you some questions about cardiovascular diseases.* | | | |
| 71 | How much do you know about “heart disease”?  Nothing at all.  You have only heard the term before but know nothing more.  You know a little about heart disease  You are very familiar with it. | \| Nothing at all \| I have only heard the term before \| I know a little about the disease \| I am very familiar with it \| \| --- \| --- \| --- \| --- \| \| 1 \| 2 \| 3 \| 4 \| | CD1K |
| 72 | And what about “stroke”? How much do you know about this disease?  Nothing at all.  You have only heard the term before but know nothing more.  You know a little about stroke  You are very familiar with it. | \| Nothing at all \| I have only heard the term before \| I know a little about the disease \| I am very familiar with it \| \| --- \| --- \| --- \| --- \| \| 1 \| 2 \| 3 \| 4 \| | CD2K |
| *Cardiovascular diseases are a group of diseases that affect the heart and brain, and their blood vessels. It includes heart attacks, where a part of the heart muscle is injured and strokes where the brain is injured.* | | | |
| 73 | In general, do you think cardiovascular diseases are becoming more or less common in Mongolia? | More1  Less2  Don’t know 3 | CD3K |
| 74 | Are you concerned about developing cardiovascular diseases yourself?  Please answer either no, yes moderately or yes very. | \| Not at all \| Yes, moderately \| Yes, Very \| \| --- \| --- \| --- \| \| 1 \| 2 \| 3 \| | CD4A |
| *The next question will ask you about things someone might do in their everyday life or things about a person that makes them more likely to get heart disease.* | | | |
| 75 | I will read 5 things, one at a time. Please tell me if you think they would increase someone’s chances of getting cardiovascular diseases or not.  It is ok if you are unsure. | \|  \| Yes \| No \| Unsure \| \| --- \| --- \| --- \| --- \| \| Smoking \| 1 \| 2 \| 3 \| \| Stress \| 1 \| 2 \| 3 \| \| Being overweight \| 1 \| 2 \| 3 \| \| Older age \| 1 \| 2 \| 3 \| | CD5K |
| *Next, please answer true, false or I don’t know* | | | |
| 76 | People with high blood pressure are more likely to have a stroke. | True 1  False 2  I don’t know 3 | CD6K |
| *Finally.* | | | |
| 77 | Cardiovascular diseases can be prevented.  *Do you agree, disagree, or not know?* | \| Agree \| Unsure \| Disagree \| \| --- \| --- \| --- \| \| 1 \| 2 \| 3 \| | CD7K |

| 1. **Knowledge, attitudes and practices related Breast and cervical cancer (BCC)** | | | | | |
| --- | --- | --- | --- | --- | --- |
| **Question** | | **Response** | | |  |
| ***(This section is only to be answered by women)***  ***(Women aged less than 30 go straight to final section)***  *I would now like to focus on another group of diseases, cancers. First, breast cancer or cancer that affects the breasts.* | | | | | |
| **Breast cancer** | | | | | |
| 78 | How much do you know about breast cancer?  Nothing at all.  You have only heard the term before but know nothing more.  You know a little about breast cancer  You are very familiar with it. | \| Nothing at all \| I have only heard the term before \| I know a little about the disease \| I am very familiar with it \| \| --- \| --- \| --- \| --- \| \| 1 \| 2 \| 3 \| 4 \| | | | BCC1K |
| 79 | Do you worry that breast cancer can affect you or your family?  *(If yes, ask “how often do you worry about it, sometimes or often?”)* | \| No, Not at all \| Yes, sometimes \| Yes, often \| \| --- \| --- \| --- \| \| 1 \| 2 \| 3 \| | | | BCC2A |
| *Now I am going to give you two statements. Please tell me if the following is true, false or you don’t know.* | | | | | |
| 80 | Finding breast cancer early means you have a better chance of becoming well again. | True 1  False 2  I don’t know 3 | | | BCC3K |
| 81 | It is possible for women to look forearly signs of breast cancer in their own breasts, by self-examination. | True 1  False 2  I don’t know 3 | | | BCC4K |
| *Breast self-examination is a method used where women such as yourself can check their own breasts for signs of breast cancer without needing to see a doctor.* | | | | | |
| 82 | Do you know how to examine your own breasts for abnormality or signs of breast cancer?  *(to be addressed only to women)* | Yes 1  No 2 | | | BCC5K |
| *Regarding breast testing, please answer yes or no to the following questions.* | | | | | |
| 83 | During the last 3years did you:  *(to be addressed only to women, if they are unsure then answer no)* | Have your ever self-examined your own breasts?  Had a physical examination of your breasts by a health worker | Yes 1  Yes 1 | No 2  No 2 | BCC6P |
| **Cervical Cancer** | | | | | |
| Now I would like to ask you some more questions about cancers. | | | | | |
| 84 | How much do you know about cervical cancer?  Nothing at all.  You have only heard the term before but know nothing more.  You know a little about breast cancer  You are very familiar with it. | \| Nothing at all \| I have only heard the term before \| I know a little about the disease \| I am very familiar with it \| \| --- \| --- \| --- \| --- \| \| 1 \| 2 \| 3 \| 4 \| | | | BCC7K |
| Cervical cancer is a cancer that affects females. It is an unhealthy growth or cancer of the female genital part called the cervix. A pap smear is a test doctors use that looks for this disease. | | | | | |
| 85 | Do you know how often it is recommended for adult women in Mongolia to have a pap smear? | Yearly 1  Every three years 2  Every 5 years 3  I don’t know 4 | | | BCC8K |
| 86 | During the last 3 years, have you had a pap-smear? | No 1  Yes 3 (skip next) | | | BCC9P |
| 87 | If not, what was the main reason you didn’t have a pap smear?  Please choose one:  (List) | I don’t have time 1  I didn’t know I needed to 2  It is expensive 3  I don’t know where to go 4  It is embarrassing 5  Others (specify)____________________6 | | | BCC10P |
| 88 | Do you worry that cervical cancer can affect you or your family?  *(If yes, ask “how often do you worry about it, sometimes or often?”)* | \| No, Not at all \| Yes, sometimes \| Yes, often \| \| --- \| --- \| --- \| \| 1 \| 2 \| 3 \| | | | BCC11A |
| *Finally.*  ***(to be addressed by ALL women)*** | | | | | |
| 89 | Do you have female children under14 years of age? | Yes 1  No 2 (skip next) | | | BCC12 |
| Ok. Now I would like to ask you about childhood vaccinations, which can prevent many diseases. | | | | | |
| 90 | Do you know if cervical cancer can be prevented by vaccine? | Yes, it can 1  No, it cannot 2  I don’t know 3 | | | BCC13K |

| **. Knowledge, Attitudes and Practices Related to Diabetes Mellitus type 2** | | | | | | |
| --- | --- | --- | --- | --- | --- | --- |
| **Question** | | | **Response** | | |  |
| *Next, In this next part, I will ask you some important questions about diabetes.* | | | | | | |
| 91 | How much do you know about diabetes?  Nothing at all.  You have only heard the term before but know nothing more.  You know a little about diabetes  You are very familiar with it. | | \| Nothing at all \| I have only heard the term before \| I know a little about the disease \| I am very familiar with it \| \| --- \| --- \| --- \| --- \| \| 1 \| 2 \| 3 \| 4 \| | | | D1K |
| *Regarding diabetes.* | | | | | | |
| 92 | Please answer the following statements about diabetes with true, false or I am unsure:  (Questions can be repeated twice) | | \|  \| True \| False \| I don’t know \| \| --- \| --- \| --- \| --- \| \| Diabetes is when there is too much sugar in the blood \| 1 \| 2 \| 3 \| \| Diabetes cannot cause loss of sensation in your feet \| 1 \| 2 \| 3 \| \| Even if I have diabetes, I can live a normal life \| 1 \| 2 \| 3 \| \| Diabetes does not damage your heart \| 1 \| 2 \| 3 \| \| Diabetes can causes blindness \| 1 \| 2 \| 3 \| \| Diabetes cannot be prevented \| 1 \| 2 \| 3 \| \| You can feel normal and healthy, but still have diabetes \| 1 \| 2 \| 3 \| | | | D2K |
| *Regarding diabetes. There are many things that people do in everyday life that increase their chance of getting diabetes. Also, though there are things people can do to reduce their chances of getting diabetes.* | | | | | | |
| **93** | Can you think of things a person can do to reduce their chances of getting diabetes?  If answer is “no”, then ask, “is this because you don’t know or there is nothing a person can do”? | | Improving their diet 1  Taking medications 2  Doing more exercise 3  Losing weight 4  Quit smoking 5  I don’t know 6  There is nothing someone can do 7  Others (specify)_____________________8 | | | D3K |
| 94 | Has a health worker ever spoken to you about how you can prevent diabetes? | | No 1  Yes 2  Don’t know/Don’t remember 3 | | | D4P |
| *That is all on diabetes.* | | | | | | |
| **7. Knowledge, attitudes and practices related Road Traffic Injuries (RTI)** | | | | | | |
| **Question** | | | | **Response** | **WT** | |
| *Every year, there are many road accidents in Mongolia. These involve cars, buses and pedestrians. I would like to ask you some questions about road accidents.* | | | | | | |
| 95 | | In this past week, how safe did you feel when you drove or walked on Mongolian roads?  Very unsafe, unsafe, safe or very safe? | | \| Very unsafe \| Unsafe \| Safe \| Very  safe \| \| --- \| --- \| --- \| --- \| \| 1 \| 2 \| 3 \| 4 \| | RTI1A | |
| 96 | | I would now like to talk about the things that might cause or contribute to accidents on Mongolian roads.  For each of the following, please rate how dangerous you think these are for Mongolian road-users.  Choose either not dangerous, moderately dangerous, dangerous or very dangerous.  (Ok to repeat last)  What about: | | \| Factor \| Not dangerous \| Moderately dangerous \| Dangerous \| Very dangerous \| \| --- \| --- \| --- \| --- \| --- \| \| Speeding drivers \| 0 \| 1 \| 2 \| 3 \| \| Alcohol use by the driver \| 0 \| 1 \| 2 \| 3 \| \| Ignoring road rules \| 0 \| 1 \| 2 \| 3 \| \| Not wearing seat belts \| 0 \| 1 \| 2 \| 3 \| \| Poor road quality \| 0 \| 1 \| 2 \| 3 \| \| Old cars \| 0 \| 1 \| 2 \| 3 \| \| Driving whilst using the phone \| 0 \| 1 \| 2 \| 3 \| | RTI2A | |
| *A few more questions on road safety.* | | | | | | |
| 97 | | In general, Mongolians rarely use a seatbelt when driving or riding in cars. Please choose the most appropriate reason for this: | | They don’t know it is unsafe to not wear seatbelts 1  Working seatbelts are often not available 2  They don’t want to wear them 3  The laws about seatbelts are not enforced 4 | RTI3A | |
| 98 | | Talking about mobile phones. Is it common for Mongolians to talk or text message whilst driving? | | Yes 1  No 2 | RTI4P | |

| **8. HOUSEHOLD CHARACTERISTICS (HC)** | | | | |
| --- | --- | --- | --- | --- |
| Question | | Response | | **WT** |
| This brings us to the final section of the questionnaire. I would like to ask you some questions about yourself, your house and your employment. | | | | |
| 99 | Firstly, your main dwellings where you live most of the time.  Does it have any of the following: answer yes or no? | \|  \| **Yes** \| **No** \| \| \| --- \| --- \| --- \| --- \| \| [A] Electricity from grid \| 1 \| 2 \|  \| \| \| [B] Electricity from generator \| 1 \| 2 \|  \| \| \| [C] Television \| 1 \| 2 \|  \| \| \| [D] Fixed-line telephone \| 1 \| 2 \|  \| \| \| [E] Refrigerator \| 1 \| 2 \|  \| \| \| [F] Computer \| 1 \| 2 \|  \| \| \| [G] Running water \| 1 \| 2 \|  \| \| \| [H] Flushing toilet \| 1 \| 2 \|  \| \| \| [I] Radio \| 1 \| 2 \|  \| \| | | HC1 |
| 100 | Next, does anyone in your main dwelling own any of the following items? Again, yes or no? | \|  \| **Yes** \| **No** \| \| --- \| --- \| --- \| \| [A Wristwatch or clock \| 1 \| 2 \| \| [B] Mobile phone \| 1 \| 2 \| \| [C] Bicycle \| 1 \| 2 \| \| [D] Motorcycle \| 1 \| 2 \| \| [E] Animal drawn cart \| 1 \| 2 \| \| [F] Car/truck \| 1 \| 2 \| \| [H] Tractor \| 1 \| 2 \| | | HC2 |
| 101 | What is the type of your main dwelling? | Apartment 1  House 2  Dormitory 3  Ger 4  ___________________Other 5 | | HC3 |
| 102 | Finally about your main dwelling, how long have you lived at this current location? | Less than 1 year 1  1-2 year 2  3-5 years 3  >5 years 4 | | N |
| **Demographic Information (DI)** | | | | |
| **Question** | | **Response** | |  |
| Next, and the final section of the questionnaire. | | | | |
| 103 | Sex (*Record Male / Female as observed)* | Male | 1 | DI1 |
|  |  | Female | 2 |  |
| 104 | How old are you? | Years | └─┴─┘ | DI2 |
| 105 | What is the **highest level of education** you have completed so far? | No formal schooling 1  Less than primary school 2  Primary school completed 3  Secondary school completed 4  High school completed 5  College completed 6  University completed 7  Post graduate degree 8  Refused 9 | | DI3 |
| 106 | Which of the following best describes your **main work** status over the past 6 months? | Government employee 1  Non-government employee 2  Self-employed 3  Non-paid 4  Student 5  Homemaker 6  Retired 7  Unemployed (able to work) 8  Unemployed (unable to work) 9  Refused 10 | | DI4 |
| *That concludes out survey. Thank you for your time and before I leave, do you have any questions, comments or concerns?* | | | | |
|  | | | | |
| *Interviewer/supervisor notes*: Use this space to record notes about the interview with this household, such as call-back times, incomplete individual interview forms, number of attempts to re-visit, etc. | | | | |
|  | | | | |
